# Extragroup interactions are not related to cognitive performance in a group-living passerine

**DOI:** 10.1098/rsos.260901

**Published:** 2026-10-28

**Authors:** Grace Blackburn, Benjamin J. Ashton, Alex Thornton, Amanda R. Ridley

**Affiliations:** ^1^ School of Biological Sciences, The University of Western Australia, Perth, Western Australia, Australia; ^2^ College of Science and Engineering, Flinders University, Adelaide, South Australia, Australia; ^3^ School of Natural Sciences, Macquarie University, Sydney, New South Wales, Australia; ^4^ Centre for Ecology and Conservation, University of Exeter, Penryn, Cornwall, UK

**Keywords:** cognition, sociality, intergroup, conflict, group-living, wildlife

## Abstract

Sociality, in particular group-living, can present many challenges to individuals and is widely thought to influence the development and evolution of cognition. However, support for the effect of sociality on cognition is mixed, and it has recently been suggested that an entire axis of sociality has been overlooked—extragroup interactions. Extragroup interactions (those that occur with a heterospecific or conspecific individual outside of the focal individual's group) present informational challenges that may favour certain cognitive phenotypes, such as discrimination, assessment and categorization. We quantified the rates of two types of extragroup interactions (intergroup and interspecific interactions) in adult Western Australian magpies (*Gymnorhina tibicen dorsalis*) and compared these to individual performance across a battery of four cognitive tasks. We also investigated whether response to playback of intruding conspecifics (an assessment of quantity discrimination abilities) was related to intergroup interaction rate. We hypothesized that greater involvement in extragroup interactions would be positively related to cognitive performance; however, we found no support for this. We did identify a relationship between intergroup interaction rate and response to playback, with individuals involved in more intergroup interactions exhibiting a greater difference in response to one and three calling extragroup conspecifics. Our findings suggest that extragroup interactions experienced in adult life do not influence performance in food-based cognitive tasks, but research on individuals during the developmental stage is needed to rule out an effect of extragroup interactions on cognitive development.

## 1. Introduction

Cognition, defined as the mechanisms through which animals receive, process, store and act on information from the environment, is essential for many aspects of life [1]. The social intelligence hypothesis, one of the prevailing hypotheses for the evolution of cognition, posits that the demands of living in social systems create challenges that select for advanced cognitive abilities [2–5] and hence predicts a positive relationship between cognition and sociality. Various social interactions tend to occur more frequently in group-living species compared to pair-bonded or solitary living species—for example the need to form and maintain multiple relationships, coordinate with group members, maintain dominance hierarchies and assess the quality of rivals and mates [2,6,7]. These social challenges are thought to influence both the evolution [2–4] and development [6,8] of the cognitive traits involved in these social challenges. Accordingly, positive links between aspects of intragroup sociality and various measures of neuroanatomy or cognitive performance have been reported across a broad range of taxa, in both comparative and intraspecific studies [9–17], including in the study species of this research, the Western Australian magpie [6,18]. However, several studies have also reported contradictory findings. For example, a number of intraspecific studies have found either no effect of sociality on cognitive performance [19–22] or a negative effect of sociality on cognition [23,24]. Recent phylogenetic analyses also suggest that large brains are more strongly associated with diet and other environmental factors than with sociality [25–28]. Consequently, whether and how social life influences cognition remains highly debated.

It has recently been suggested that one aspect of sociality—interactions with extragroup outsiders (comprising both intergroup and interspecies interactions)—has been overlooked as a factor influencing individual cognition [29–31]. First proposed as a driver of cognition in humans in 1989 [32], intergroup interactions are common in many species and may present a suite of additional cognitively demanding challenges [29,33]. The need to track extragroup third-party relations [34], assess secondary cues (e.g. faeces of extragroup individuals), eavesdrop on extragroup conspecifics [35–37] and participate in intergroup interactions [38] are aspects of extragroup sociality that may entail cognitive demands. Intergroup contests in particular may involve multiple information processing (i.e. cognitive) demands. Numerous factors may influence whether an individual enters into a contest: the ownership and value of the contested resource (i.e. food, mate or territory) [39], individual status or incentives [40,41], the need to protect offspring [42] and the resource holding potential (RHP) of both their opponent and themselves [38,39,43–45]. Prior to investing in an intergroup interaction, individuals may therefore need to assess and weigh up the potential costs and benefits of the interaction, an assessment that may draw on multiple cognitive abilities, including discrimination, learning, memory and decision-making. For example, quantity discrimination may be important for group-living individuals when assessing the RHP of their opponents, as the RHP of a group is probably determined by the number of individuals within that group [46,47]. Similarly, individuals may benefit from the ability to discriminate between different classes of extragroup individuals (e.g. ‘neighbours’ versus ‘strangers’—the ‘dear enemy’ effect [48–50]) before responding. Once involved in a contest, individuals may gain an advantage by being able to continually assess the behaviour and quality of their rival(s), as well as the number of actively participating opponents, and flexibly update and adjust their own behaviour in response [22,51,52]. Likewise, individuals may benefit from the ability to observe and remember the cues of conspecifics they are interacting with [29,38,45]. In some ways, extragroup interactions may be more cognitively challenging than intergroup interactions, as opportunities to gather information about extragroup individuals may be rarer, occur over shorter time windows and entail higher costs [53,54]. Although interest in the cognitive consequences of sociality has grown in recent decades [5,6], very little research has been conducted to elucidate the relationship between cognition and intergroup interactions, despite such interactions being common and likely to involve several cognitive skills.

As well as intergroup interactions within species, interactions between species are common and probably present cognitively demanding challenges that share similar underlying cognitive mechanisms to intraspecific interactions [31,36,55–57]. While the effects of interspecies mutualisms [58–60] and predator–prey interactions [55,61–65] on cognition have garnered attention, there are many other interspecific interactions that may influence cognition. For example, eavesdropping on heterospecific alarm calls, which is hypothesized to involve learning [36,66,67], can help to enhance survival via a more accurate anti-predator response and therefore may select for individuals more adept at interpreting and responding appropriately to the alarm calls of other species [36,37,67–69]. Species occupying similar ecological niches may also interact aggressively when competing for resources such as food or territory [31]. In such cases, interspecific aggression may be as costly as intraspecific aggression and therefore probably imposes similar selection pressures on the behavioural and cognitive mechanisms underlying such aggressive interactions [31]. The occurrence of kleptoparasitism by species, such as the fork-tailed drongo (*Dicrurus adsimilis*), represents a cognitive challenge both for the kleptoparasite, who must assess how and when to launch an attack to gain the most benefit [70,71], and for the host, as they may benefit from learning to adapt to kleptoparasitic threats [72]. Brood parasitism can present another example of a cognitively challenging interspecific interaction [31,73]. For hosts, the ability to maintain vigilance against brood parasites, as well as to recognize and count their own eggs [74,75], may be important defences against brood parasitism that can result in increased reproductive success and is probably underpinned by cognition [76].

To date, the potential effects of intergroup and interspecific interactions on cognitive performance have been largely overlooked, both theoretically [29,32,65] and empirically [30]. To our knowledge, the only study to have investigated this relationship in non-human animals, a comparative interspecific analysis across 104 group-living species of primates, found a significant positive relationship between home-range overlap (used as a proxy of intergroup conflict) and endocranial volume (used as a proxy for cognition) [30]. While the use of brain size as a proxy of cognitive is often criticized, particularly in comparative analyses [77–79], findings from this study nevertheless provide support for the hypothesis that extragroup aspects of the social environment, such as intergroup interactions, may be related to cognition. However, whether extragroup sociality can help explain *intraspecific* variation in cognitive performance has not yet been considered.

We suggest that exposure to both intergroup and interspecific interactions presents cognitively demanding challenges to individuals and should therefore be positively related to performance in cognitive tasks representing traits likely to be involved in extragroup interactions. To investigate this, we combined observations of extragroup interaction occurrence with cognitive testing and playback experiments in wild adult Western Australian magpies. The Western Australian subspecies of Australian magpie (hereafter, magpie) is a highly territorial, group-living bird that regularly interacts with extragroup conspecifics in high-risk territorial disputes [80–82], as well as in extragroup forays for mating opportunities [82]. Interactions with extragroup conspecifics can vary significantly in nature, from aggressive physical contests to tolerance and even intergroup mating [80–82]. Interactions between magpie groups are often characterized by visual and vocal displays [81,83], with individuals carolling (the main territorial song of this species [83,84] or chorusing together with other group members [83,85]. Magpies also regularly interact with heterospecifics, including joining other species at mobbing events of predators or threats, foraging in mixed species flocks [86] and competing with heterospecifics for food and other resources [87–89]. Magpies have also been observed responding to heterospecific mobbing calls from noisy miners (*Manorina melanocephala*), magpie larks (*Grallina cyanoleuca*) and crimson rosellas (*Platycercus elegans*) [35,37]. The frequent occurrence and conspicuous nature of both intergroup and interspecific interactions in this species allow for investigation into the relationship between extragroup interactions and cognition.

Previous research on magpies has found a positive relationship between intragroup sociality (measured as group size) and general cognitive performance (a measure encompassing four cognitive tasks in the physical domain: associative learning, reversal learning, spatial memory and inhibitory control) [6]. More recently, social network analyses revealed that cognitive performance in an associative learning task was related to intragroup aggression, with individuals receiving aggressive interactions more frequently performing better and those initiating aggressive interactions more frequently tending to perform worse in this task. Additionally, individuals that were involved in aggressive interactions with more group members exhibited lower cognitive performance [18]. Individuals receiving more aggressive interactions may have an increased requirement to form associations between cues that precede an aggressive interaction (e.g. vocal or postural cues) and the interaction itself. Alternatively, they may relate to a trade-off between personality and cognition (such as speed–accuracy trade-offs often seen in bolder individuals) or heightened stress levels in aggressive individuals. Regardless of the mechanism behind this, these findings reveal an important influence of sociality on cognition in magpies and make this an ideal study system for this research. We investigated whether extragroup sociality (measured as intergroup and interspecific interaction rates) was related to cognitive performance in adult magpies. To do this, we presented magpies with a battery of cognitive tasks representing skills likely to be important for extragroup sociality. Since previous research found evidence that physical and social cognition are not aligned in Western Australian magpies and instead represent two separate cognitive domains [90], our cognitive battery comprised tasks representing cognitive traits from both the physical domain (traits thought to have evolved in the context of foraging and related to navigating space, understanding causality and discriminating between quantities [91]) and the social domain (traits considered to have evolved owing to the social challenges of group-living, related to communication, social learning and theory of mind [91,92]). The social task used in this study was an observational spatial memory task, while the three physical tasks used were associative learning, spatial memory and quantity discrimination. The three physical tasks have all previously been related to various measures of intragroup sociality in this species [6,18,93,94] and test traits likely to be used in extragroup interactions (see §2 ‘Methods’ for more details). We hypothesized that magpies involved in more intergroup interactions would exhibit higher cognitive performance across a battery of four cognitive tasks representing abilities likely to be important in extragroup interactions. In addition, as food-based cognitive tasks may not be ecologically relevant to intergroup contests, we presented magpies with playbacks of different numbers of callers, as a more relevant measure of quantity discrimination abilities. Birds were presented with territorial songs of one extragroup individual (represented by a single carol) and three extragroup individuals (represented by a single chorus of three birds singing simultaneously), vocalizations that magpies would commonly hear in an intergroup contest. The behavioural and vocal response of the focal bird was then quantified to determine if intergroup interaction rate was related to the ability of individuals to discriminate between the songs of one and three calling individuals. We hypothesized that birds involved in more intergroup interactions would be more able to discriminate between the songs of one and three calling extragroup conspecifics, owing to them having more experience in these interactions, compared to birds that are less frequently involved in intergroup interactions.

## 2. Methods

### 2.1. Study species and site

Magpies are large (250–400 g) group-living passerines found in urban parklands and open grassy areas in the southwest of Western Australia [95]. This subspecies lives in stable territorial groups of 2–12 individuals [96]. Individuals within groups interact frequently, exhibit complex communication and cooperation and defend year-round territories [18,96]. Magpies frequently participate in intergroup interactions with neighbouring groups for resources such as territory, food and mating opportunities [82,96,97]. Magpies are sexually dichromatic, with sex visually discernible from three years of age when individuals achieve their full adult plumage [83,95].

The study population consisted of 110 magpies within 16 groups located in the urban suburbs of Crawley and Guildford, Perth. This population is habituated to humans, and most individuals are colour-ringed for individual identification [6]. Unringed individuals in the population are identifiable by plumage anomalies or scarring. This population experiences low rates of dispersal, with an average (± s.e.) dispersal age of 2.47 ± 0.42 years [98]. All fieldwork for this study was conducted between July 2021 and May 2023 between 05.00 and 11.00, when magpies are most active [99]. During these years, the sex ratio of the population (calculated by dividing the number of males in the population by the number of females) was slightly female biased (2021, sex ratio = 0.78; 2022, sex ratio = 0.90; 2023, sex ratio = 0.89), as has been previously reported for this population [96]. Magpies typically become reproductively active at 3–4 years of age [96]. The breeding season spans from August to December [95,96], and magpies may attempt multiple broods per season (A. R. Ridley, G. Blackburn 2021–2023, personal observation).

### 2.2. Behavioural focals

To quantify the rate of intergroup and interspecific interactions that individuals were involved in, we conducted both individual- and group-level observational focals. Focals were recorded using customized ethograms created in CyberTracker [100]. Individual focal observations consisted of 20-minute time activity periods where all behaviours that the focal magpie was exhibiting were recorded, including the occurrence of any intergroup or interspecific interactions. In total, we conducted 471 20-min individual focals across 89 adult individuals from 16 groups (mean ± s.e. = 5.29 ± 0.28 focals per individual). During group-level focals (which ranged from 15 to 60 min, mean focal length = 31 min), we noted the presence of individuals within the group (presence/absence of individuals was noted every 5 minutes), as well as the occurrence of any interactions of those individuals with other magpie groups or with other species, and the type of interaction that occurred. In total, we conducted 5945 min of group-level focals across 16 groups (mean ± s.e. = 371.6 ± 12.3 min per group).

Intergroup interactions were considered to have started when two different groups, or individuals from two different groups, came within 30 metres of each other. This threshold of 30 metres was chosen as it is typically the maximum distance at which discernible intergroup interactions can be observed and recorded. Where possible, for each intergroup interaction, we noted which individuals from each group were participating, as well as the length of the intergroup interaction. Intergroup interactions were considered to have ended when the two groups were more than 30 metres apart, had returned to normal behaviours (perching or foraging, and non-vigilant) and had not vocalized for at least 2 minutes. Intergroup interactions included tolerant interactions, vocal interactions and physical aggressive interactions. Tolerant interactions occurred where groups were less than 30 metres apart but were not investing in physical or vocal displays. Vocal interactions were defined as when two groups were engaged in a vocal exchange (chorusing back and forth between groups) less than 30 metres apart. Physical interactions were defined as when individuals from different groups chased and/or physically fought each other (which always occurred within 30 metres of each other).

Interactions between focal magpies and other species were recorded as they occurred, including species identification, and the type of interaction that occurred. Magpies regularly interact with heterospecifics at our study site, including red wattlebirds (*Anthochaera carunculata*), Australian ravens (*Corvus coronoides*), magpie larks (*G. cyanoleuca*) and laughing kookaburras (*Dacelo novaeguineae*). Interspecific interactions included magpies chasing, mobbing or swooping other species (classified as magpie aggressive interactions), and magpies being chased or swooped by other species (classified as interspecific aggressive interactions). Each type of interaction rate for each individual was calculated by combining both individual and group-level focals and dividing the number of instances of that interaction by the total time that individual was observed for. In total, we collected 427.57 hours of individual behavioural observations from 89 adult magpies belonging to 16 groups (mean ± s.e. = 4.80 ± 0.17 observation hours per magpie).

### 2.3. Cognitive testing

Magpies were presented with a battery of four cognitive tasks (figure 1), chosen to quantify a range of traits likely to be important in social interactions:

— associative learning, an individual's ability to learn predictive contingencies between environmental cues [101], is a well-studied cognitive trait thought to play a role in multiple contexts, including interactions with (and recognition of) conspecifics [29,38,101,102]. Individuals may benefit from the ability to learn and store information from previous interactions and use this to inform future behaviour [22,29,38];
— spatial memory is hypothesized to be important for social interactions, particularly in territorial species, as it allows individuals to remember the location of territory borders, as well as the location of previous territorial disputes, and neighbouring groups that they may frequently interact with [29,103–107];
— quantity discrimination is widely considered to be important for both interactions with conspecifics, particularly in contest scenarios where the ability to assess the number of competitors relative to the number of defenders can be beneficial [43,45,47,52,108,109], as well as with heterospecifics, as quantity discrimination is thought to play a role in the collective anti-predator mobbing response of individuals [110–112]; and
— observational spatial memory, the ability to observe and remember the behaviour of others, is considered to be a good representation of the ability to observe, remember and appropriately respond to the cues of others and is therefore an important cognitive skill for group-living species [113–115] and for individuals engaged in interactions with other conspecifics or groups.

**Figure 1.**
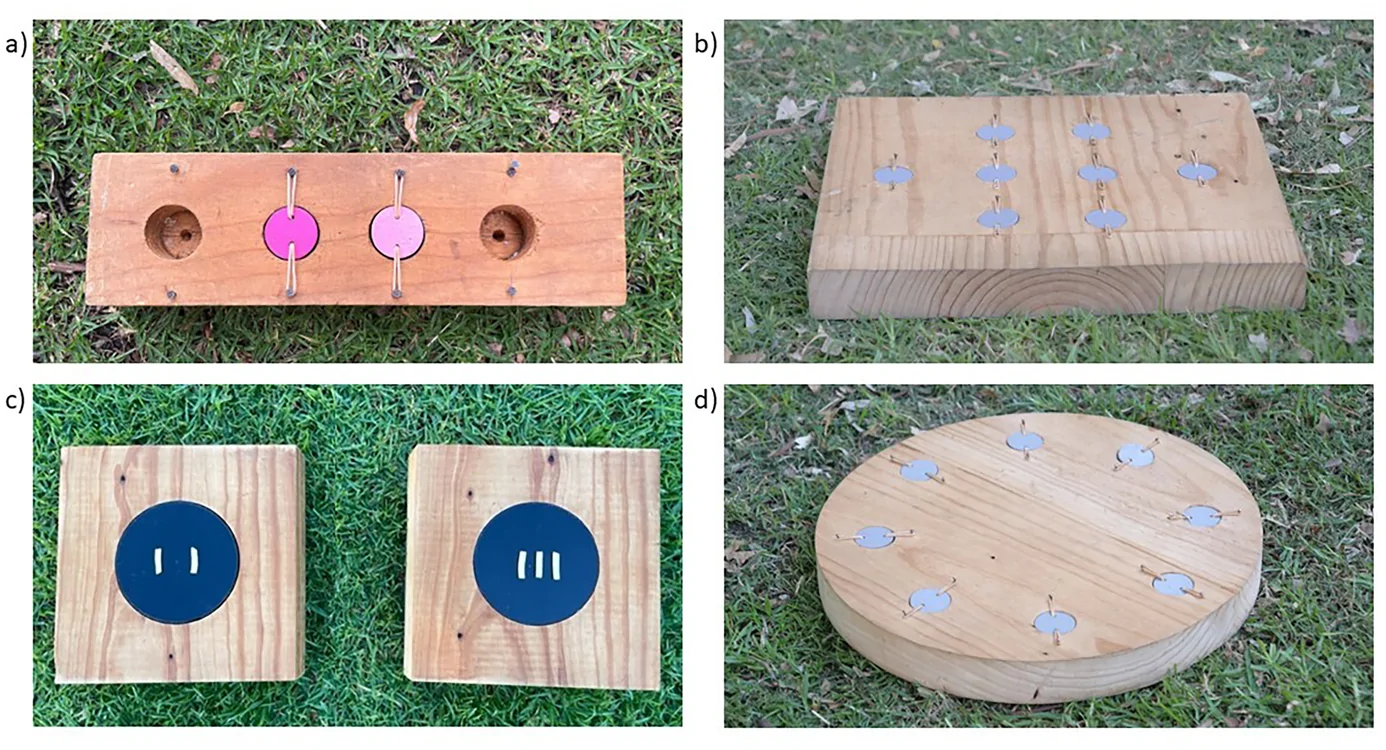
Battery of four cognitive tasks used in this study: (a) the associative learning task, (b) spatial memory task, (c) quantity discrimination task, and (d) observational spatial memory task.

All cognitive tests used mozzarella cheese as a food reward (mozzarella cheese has been previously used as a food reward for cognitive testing in this species [5,6]), and other than the quantity discrimination task (where food rewards were placed on top of the task for magpies to see the number ratios), all tasks required individuals to peck downwards at a lid to access the food reward. Magpies are terrestrial foragers, meaning this pecking movement resembles their natural foraging behaviour [99]. Cognitive testing was conducted only when focal individuals were socially isolated (other magpies were at least 10 metres away [6,80], to minimize the potential confounding effects of social learning on performance) and were not displaying signs of vigilance.

#### 2.3.1. Associative learning

To quantify associative learning performance, we used a task that required individuals to learn an association between a colour shade and a food reward. The task consisted of a rectangular wooden block (31 × 9 × 4 cm: length, width, depth) with four circular wells drilled into it, two of which were covered with plastic PVC lids painted different shades of the same colour (different shades of the same colour were used to avoid any potential confounding effects of a colour bias [116–118]). These lids were held in place by elastic bands that allowed them to swivel on an axis when pecked so birds could access the food reward beneath them. To prevent birds from using olfactory cues to locate the food reward, the inside of each well was rubbed with mozzarella cheese prior to the start of testing. The study population has previously been tested on causally similar but visually distinct variations of this task in the past [6], and thus, we only tested birds on colours they had not previously seen. For each individual, we randomly chose a colour shade (either dark or light) to be the rewarded shade.

Testing followed the protocol of Shaw *et al.* [119] and Ashton *et al.* [6]. In the first trial, individuals were allowed to peck at and search both wells to determine that only one well contained a food reward. In all subsequent trials, birds were only allowed to peck one well before the task was removed. The side that the rewarded well was on was randomly rotated between trials to ensure birds were not associating the side of the task with the food reward. We considered individuals to have passed the task when they pecked the rewarded lid first in 10 out of 12 consecutive trials [80,93,119]. The number of trials individuals took to reach this criterion was their associative learning score (higher scores reflect worse task performance as they indicate that individuals took longer to learn the association).

#### 2.3.2. Spatial memory

To quantify spatial memory performance, we used a wooden foraging grid (36 × 40 × 4.5 cm^3^) with eight wells covered with light grey plastic PVC lids. Prior to testing, wells were rubbed with mozzarella cheese to ensure birds were not using olfactory cues to locate the food reward. Testing followed the protocol of Shaw *et al.* [119] and Ashton *et al.* [6], but with an additional testing phase. One well was randomly selected for each individual to be the rewarded well throughout all stages of testing. Testing consisted of three stages (and six trials in total): (i) the training stage, (ii) the testing stage, and (iii) the probe stage. In the training stage, birds were presented with the spatial memory task and allowed to search all wells on the foraging grid until they located and consumed the food reward. The foraging grid was then removed and rebaited before this training trial was repeated after 5 minutes. The testing stage of the experiment consisted of three presentations of the foraging grid 24-, 48- and 72 hours after the initial training stage. The 24- and 48-hour presentations of the spatial memory task followed the protocol of Shaw *et al.* [119] and Ashton *et al.* [6], and a 72-hour presentation was added because it has been previously found that spatial memory performance improves over time or with the number of trials [6,120]. We quantified spatial memory performance as the combined total number of wells searched prior to finding the food reward (including revisits to the same well) in the 24-, 48- and 72-hour presentations of the task (*sensu* [6,119]).

The final presentation of the spatial memory task was conducted 5 minues after the 72-hour presentation of the task and consisted of a single ‘probe’ trial that was included to confirm individuals were not using olfactory cues to locate the food reward. The spatial memory task was rotated 180 degrees (out of sight of the focal individual) and presented to the bird without any well containing a food reward. Therefore, while the grid appeared the same to the focal bird, the previously rewarded well was now on the opposite side of the grid. The number of wells that the bird searched before finding the well that was in the rewarded position was considered their olfactory probe score. If the focal bird was using olfactory cues rather than spatial memory to locate the food reward, then they would be expected to search the previously baited well (now on the opposite side of the grid), and hence their olfactory probe score would be significantly different from their 72-hour score. If birds were using spatial cues, they should instead search the well that was now in the rewarded position, and their olfactory score would be similar to their 72-hour score.

#### 2.3.3. Quantity discrimination

To quantify quantity discrimination performance, we used two identical wooden boards (20 × 20 × 4.5 cm^3^) that each had a large well (10 cm diameter, 3 cm deep) drilled into it. The wells were covered with black lids that were immovable to magpies. Wells were filled with mozzarella cheese to help control for olfactory cues and ensure birds were selecting a board based on the strands of cheese presented on top of it and not using olfactory cues to select a board. We presented strands of mozzarella cheese, cut identically to 2.5 mm length, on the black lids of the wooden boards, in four different ratios: 2 : 2 (a control to test for the presence of a side bias), 2 : 3, 2 : 4 and 2 : 5. Black lids were identically marked with six small lines, 1 cm apart from each other, to mark where cheese strands would be placed and to ensure that the two stimuli were matched for convex hull (the width/extension of the stimuli) [121]. For each trial, cheese strands were placed on the black lids of the boards (such that they were easy for magpies to see and evaluate), and boards were placed equidistant in front of the focal bird, 1 metre from the focal bird and 30 cm from each other. The experimenter then stepped back and allowed the focal bird to approach the boards and select and eat the food reward from one board, which was considered the focal bird's choice. Birds were allowed to consume the entire food reward on the board that they first touched (and therefore were considered to have selected), and following this, both boards were removed.

Testing proceeded following the protocol of Blackburn *et al.* [90] and Hunter *et al.* [94], with each of the four ratios (2 : 2, 2 : 3, 2 : 4 and 2 : 5) presented to focal birds every day for 15 days, resulting in a total of 60 trials for each bird. Ratios were presented in a randomized order, and the side holding the greater number of cheese strands was randomly alternated to ensure birds were making their selection based on the amount of food and not a side. An individual's quantity discrimination score was quantified as the number of times they selected the smaller amount of food, such that a higher quantity discrimination score was indicative of worse performance, in line with the other cognition tasks described above (for the purposes of the principal component analysis (PCA)).

#### 2.3.4. Observational spatial memory

To quantify observational spatial memory performance, we used a circular wooden foraging grid (40 cm diameter × 4.5 cm depth) with eight wells drilled into it around the outside of the grid. Wells were covered with light grey PVC plastic lids. Prior to testing, all wells were rubbed with mozzarella cheese to control for olfactory cues. The test consisted of four trials that took place over four consecutive days, following the protocol of Blackburn *et al.* [90]. For each individual and each trial that individuals undertook, one well was randomly chosen to be the rewarded well. Each trial began with the experimenter baiting the rewarded well in view of the focal bird, while the focal bird was attentive (defined as oriented towards the task and looking at the experimenter *sensu* [122]) and within 3 metres of the task. The experimenter then stepped back, so the focal bird could approach the task and search wells until they located the rewarded well and ate the food reward. This was repeated 24-, 48- and 72 hours later, with a new well baited in each trial. We quantified the observational spatial memory score of an individual as the cumulative number of wells searched prior to locating the rewarded well (including revisits to previously searched wells) in each of the four trials.

#### 2.3.5. Proxies of motivation and environmental conditions

Individuals may vary in their performance on cognitive tasks depending on motivational factors such as hunger levels or neophobia [123,124]. We therefore measured several proxies of motivation for each task: latency to approach the task, measured as the time taken from when an individual first came within 5 metres of the task (or 1 metre from the task in the case of the observational spatial memory task, since this task required the focal bird to be closer at the start of the task, to observe where the experimenter was placing food) to when they first interacted with the task [6,118], body mass, foraging effort and foraging efficiency. Magpies have previously been trained to jump onto an electric top-pan scale for a food reward, allowing us to measure the body mass of birds in our population [6,96].

For each cognitive task (and within 4 hours of testing), we collected 10-minute behavioural focals to determine the foraging effort and foraging efficiency of individuals. We used a customized ethogram in the CyberTracker programme [100] to record the behaviour of focal birds continuously. From these focals, we calculated foraging effort as the time individuals spent foraging out of the total focal time. Foraging efficiency was calculated as the biomass (in grams) of prey consumed by the focal bird divided by the time spent foraging (in minutes). The biomass of prey consumed by focal birds was calculated based on the size of the prey relative to the size of the focal bird's bill, using biomass calculations presented in [99] (mean (± s.e.) = 3.9 (± 0.2) focals per bird, *n* = 56 birds tested).

Variation in cognitive performance can be impacted by temperature [93,125]. Therefore, all tests were conducted when temperatures were below 27°C, to ensure there were no confounding effects of heat stress on cognitive performance [93,125,126].

### 2.4. Quantity discrimination playback experiment

Naturally emitted territorial songs (carols and choruses) of known adult magpies and groups of adult magpies in our population were collected using a RODE NTG-2 directional microphone set within a blimp suspension windshield system and attached to a Roland R-07 wave/MP3 recorder, set at a sampling rate of 44.1 kHz. Songs were collected by an experimenter 1–5 m from the calling bird(s). For each song collected, we noted the identity of the individual(s) that vocalized, as well as their sex and group identity. Playbacks of one caller and three callers were then prepared using Audacity v.3.0.2. Only high-quality songs (with a high signal-to-background-noise ratio), during which no other birds could be heard, were used for playback preparation. One-caller songs consisted of a carol of a single calling bird, while three-caller songs consisted of a chorus of three calling birds singing simultaneously, as this reflected natural territorial singing behaviour. Each song was used in only one playback to avoid pseudoreplication (i.e. one-caller playback tracks consisted of 17 different songs from 13 individuals, while the three-caller playback tracks consisted of 17 different songs from different combinations of 32 individuals). Playback tracks consisted of 5 seconds of silence, followed by 2 seconds of background noise (collected when no loud anthropogenic noise or bird vocalizations were occurring in the territory of focal groups using the same set-up used for song collection) that faded in, followed by a single song of either one or three extragroup calling magpies. Vocalizations were normalized to ensure songs were broadcast at their natural amplitude [127] (approx. 81 and approx. 86 dB for one- and three-caller playback tracks, respectively, to replicate that of natural one- and three-caller songs [127]) and therefore represented cues magpies would naturally encounter in their environment. Songs were not altered other than normalization and therefore reflected natural songs that magpies would hear in their normal environment. A two-sample *t*-test revealed that three-caller songs were slightly longer (0.69 s) than one-caller songs (*t* = −3.69, *p* < 0.01; one-caller song mean ± s.e. = 3.18 s ± 0.11, three-caller song mean ± s.e. = 3.87 s ± 0.15). Playback tracks were then loaded onto a FiiO M6 portable high-resolution music player, attached by an aux cord to a UE Boom 2 speaker for playback presentations. A sound pressure level meter was then used to check that the amplitude of tracks was the same as the amplitude of naturally occurring magpie songs of one- or three callers at 1 metre from the speaker/bird.

Focal birds were each presented with two playback tracks: playback of one extragroup individual and playback of three extragroup individuals. Playbacks were presented in a randomized order and at least 5 days apart to minimize stress caused by the perception of a territorial intrusion, as well as potential habituation to the speaker. Playbacks presented to focal birds were from individuals unfamiliar to them (located ≥ 15 km from the focal bird's territory) and were presented to the focal bird in the centre of their territory. The two playback tracks presented to each focal bird consisted of different singing individuals, to minimize habituation that may arise from hearing the same individual played back multiple times. Playbacks began when the focal bird was foraging in an open area, exhibiting normal (non-vigilant) behaviour, and was in social isolation (other magpies > 10 metres away *sensu* [80]. The speaker was placed 10 metres from the focal bird, and playback tracks were started following 1 minute or more of silence (during which no other magpie vocalizations or loud noises occurred). The response of the focal bird was videorecorded using a Panasonic HC-V180 camcorder from 1 minute prior to the start of playback and was terminated 2 minutes post-playback.

Videos of the responses of focal birds to playback were analysed with the analyst (G.B.) blind to playback treatment. The following behaviours were quantified: the time focal birds spent vigilant in the 2-minute post-playback (birds were considered vigilant when displaying an erect posture and scanning the environment; [80,128]) and the number of territorial songs sung by the focal bird in the 2-minute post-playback.

### 2.5. Statistical analyses

Statistical analyses were performed in R v.4.4.2 [129]. We first ensured that magpies were using their memory (as opposed to randomly selecting wells) in both the spatial memory and observational spatial memory tasks. To do this, we compared scores in each trial to a random search expectation of 4.5 wells (from an equation derived from the negative hypergeometric distribution by [130] *sensu* [6,119]), using non-parametric two-tailed, one-sample Wilcoxon tests to compare the number of lids flipped by magpies in each trial to this random search expectation. Similarly, to ensure magpies were performing significantly better than random when selecting the larger quantity of food in the quantity discrimination task, we conducted binomial tests across each test ratio (2 : 3, 2 : 4 and 2 : 5) to determine if the number of times magpies chose the larger food reward was significantly different from random.

#### 2.5.1. Relationship between cognitive performance across cognitive tasks

Previous research on this study species found that performance across the three physical cognitive tasks presented in this study (associative learning, spatial memory and quantity discrimination) loads strongly and positively onto a first principal component (PC1; [90]). However, observational spatial memory (a task likely to tap into aspects of social cognition), is instead loaded positively and strongly onto a second principal component, suggesting that in magpies, this task does not align with the three tasks assessing physical cognition [90]. Therefore, factors affecting physical and social cognitive tasks were analysed separately. We therefore first confirmed that cognitive performance measured in this study across the three physical cognitive tasks (associative learning, spatial memory and quantity discrimination) was positively correlated and loaded onto a single principal component. To do this, we first performed a Kaiser–Meyer–Olkin (KMO) test to measure sampling adequacy and confirm that our data were suitable for factor analysis. The KMO test revealed an overall measure of sampling adequacy (MSA) of 0.62, indicating that cognitive scores across these three tasks were suitable for factor analysis [131]. We then proceeded to perform Kendall's rank correlations on the scores from each pair of tasks (supplementary material, table S1). We then performed an unrotated PCA on the scores from these three tasks using the *FactoMineR* package [132]. Results from our PCA confirmed that the three physical cognitive tasks loaded strongly and positively onto PC1, which explained 55.25% of variation in individual cognitive performance (supplementary material, table S2). We extracted the individual coordinates of PC1 and considered these a measure of general cognitive performance in the physical domain (hereafter, PCP—to see the analysis of each cognitive task separately, see the supplementary material, §4).

We tested separately how participation in intergroup and interspecific interactions relates to both social (observational spatial memory) and physical (PCP: encompassing associative learning, spatial memory and quantity discrimination) cognitive performance. We also tested how participation in extragroup interactions is related to each of the three physical cognition tasks (spatial memory, associative learning and quantity discrimination) separately (for more information, see the supplementary material, S4 and tables S7–S9).

#### 2.5.2. Model selection

We fitted linear mixed models (LMMs) using the *glmmTMB* package [133]. Continuous predictors used in analyses were scaled (means subtracted and divided by the standard deviation). All models included group ID as a random term. We conducted model selection using Akaike's information criterion corrected for small sample sizes (AICc) and only tested models that presented plausible *a priori* hypotheses [134]. Models were compared to a null model containing only the intercept and random terms. If a term when tested alone was not significant, it was not included in additive models with other terms but was still included in interaction models if these presented plausible *a priori* hypotheses. A top model set was created that contained all models with AICc values that were within 2 AICc of the top model (the model with the lowest AICc, considered the best fit). Terms in these models were only considered significant if their parameter 95% confidence intervals did not intersect zero [135,136]. If two models had similar AICc values, the model with the fewest terms was chosen as the most parsimonious [137]. The DHARMa package was used to check for normality of residuals, overdispersion and the presence of outliers [138].

#### 2.5.3. Factors explaining variation in individual cognitive performance

To investigate how the frequency of intergroup and interspecific interactions was related to cognitive performance, we fitted LMMs with observational spatial memory score and PCP as the response variables. LMMs investigating factors affecting PCP and observational spatial memory had a normal distribution. All interaction predictors were calculated at the individual level by dividing the number of interactions of each type that a focal individual was involved in by the total time that the focal individual was observed. For intergroup interaction predictors, we included vocal interaction rate (calculated as the number of vocal intergroup interactions per observation hour), physical aggressive interaction rate (calculated as the number of physical aggressive intergroup interactions per observation hour) and tolerance interaction rate (calculated as the number of tolerant intergroup interactions per observation hour), as well as a predictor of total intergroup interaction rate that combined all intergroup interaction types (calculated as the total number of intergroup interactions per observation hour) and the average length of intergroup interactions that an individual was involved in (in seconds). For interspecific interaction predictors, we included magpie aggressive interaction rate (calculated as the number of interspecific interactions where magpies were aggressive to other species per observation hour), interspecific aggressive interaction rate (calculated as the number of interspecific interactions where other species were aggressive to magpies per observation hour) and a predictor of total interspecific interaction rate that combined these two interspecific interaction types (calculated as the total number of interspecific interactions per observation hour) as explanatory terms.

We also investigated whether individual and motivational factors affected cognitive performance in both the observational spatial memory task and PCP (presented in the supplementary material, tables S4 and S6 respectively). For this analysis, we included sex, latency to approach the task, body mass, foraging effort and foraging efficiency (averaged across the three tasks included in PCP for those models looking at factors affecting PCP). We ran these analyses separately on subsets of individuals for whom we had foraging focals and body mass measures.

#### 2.5.4. Factors explaining response to quantity discrimination playbacks

Another set of LMMs was fitted to investigate if intergroup interaction rate was related to the ability of individuals to discriminate between different numbers of callers in a playback experiment, on a subset of 17 individuals that had undergone playback experiments. The response variables for these LMMs were as follows: (i) the difference in time spent vigilant following the three-caller and one-caller playback track (calculated as the time (seconds) spent vigilant in the 2 minutes following the three-caller playback track minus the time (seconds) spent vigilant in the 2 minutes following the one-caller playback track), and (ii) the difference in the number of songs sung by the focal bird in the 2 minutes following the three-caller and one-caller playback track (calculated as the number of songs sung following the three-caller playback track minus the number of songs sung following the one-caller playback track) (see the supplementary material, table S11 for results). Predictors for these models included sex and the intergroup interaction rate of the focal bird.

## 3. Results

### 3.1. Individual variation in cognitive performance

Magpies completed the associative learning task, spatial memory task and observational spatial memory tasks in an average of 17.45 trials (s.e. = 1.13, range 10–41, median = 14, *n* = 51 birds), 12.68 trials (s.e. = 0.87, range 3–25, median = 12, *n* = 56 birds) and 13.66 trials (s.e. = 0.71, range 4–27, median = 13, *n* = 56 birds), respectively. For all three of these tasks, a higher score is indicative of worse performance.

In the quantity discrimination task, magpies chose the larger amount of food more often than chance in the 2 : 3 (*n*= 705 trials on 47 magpies, *p* < 0.001; larger amount was chosen in 58% of trials), 2 : 4 (*n* = 705 trials on 47 magpies, *p* < 0.001; larger amount was chosen in 65% of trials) and 2 : 5 (*n* = 705 trials on 47 magpies, *p* < 0.001; larger amount was chosen in 72% of trials) ratios, confirming that magpies are able to discriminate between two quantities of a food reward. When combining trials of all ratios, birds selected the larger food reward on average 29.4 times out of a total of 45 trials (s.e. = 0.68, range 21–41, median = 29, *n* = 47 birds).

Magpies did not search significantly differently from the random search criterion of 4.5 wells in the 24-hour trial (mean ± s.e. = 4.04 ± 0.46, median = 2; one-sample Wilcoxon test: *V* = 618, *p* = 0.14) or the 48-hour trial (mean ± s.e. = 5.09 ± 0.48, median = 5; one-sample Wilcoxon test: *V* = 866, *p* = 0.58) of the spatial memory task. However, in the 72-hour trial, magpies searched significantly fewer wells than expected if searching randomly (mean ± s.e. = 3.55 ± 0.35, median = 3; one-sample Wilcoxon test: *V* = 429, *p* < 0.01), suggesting magpies are able to remember the location of the rewarded well at the 72-hour time point. The average number of wells searched by magpies in the olfactory probe trial was 3.81 (s.e. = 0.34, median = 3), which was not significantly different from the number of trials searched in the 72-hour trial (mean ± s.e. = 3.55 ± 0.35, median = 3; two-sample Wilcoxon test: *V* = 253.5, *p* = 0.31), suggesting magpies were not using olfactory cues to locate the rewarded well.

In the observational spatial memory task, magpies performed significantly better than the random search criterion of 4.5 wells in all trials (first trial mean ± s.e. = 2.93 ± 0.25, median = 3, one-sample Wilcoxon test: *V* = 198, *p* < 0.01; second trial mean ± s.e. = 3.36 ± 0.33, median = 3, one-sample Wilcoxon test: *V* = 383, *p* < 0.01; third trial mean ± s.e. = 4.00 ± 0.34, median = 4, one-sample Wilcoxon test: *V* = 554, *p* < 0.05; fourth trial mean ± s.e. = 3.38 ± 0.33, median = 3, one-sample Wilcoxon test: *V* = 398.5, *p* < 0.01), suggesting magpies are able to remember and locate a food reward that they observed being hidden by an experimenter.

### 3.2. Relationship between individual cognitive performance across tasks

PCA revealed that performance on the associative learning, spatial memory and quantity discrimination tasks loaded strongly and positively onto PC1 which explained 55.25% of variation in cognitive performance across tasks (supplementary material, table S2).

### 3.3. Intergroup and interspecific interactions

Magpies had intergroup interactions with conspecifics on average 0.39 times an hour (± 0.04, *n* = 89 adult magpies). The most common type of intergroup interactions was vocal interactions (44.8% of all intergroup interactions), followed by tolerant interactions (37.9% of all intergroup interactions) and then physical interactions (17.2% of all intergroup interactions) (figure 2a). Sex did not affect how often individuals were involved in intergroup interactions (Mann–Whitney test, *w* = 1066, *p* = 0.521, *n* = 89 adult magpies).

**Figure 2.**
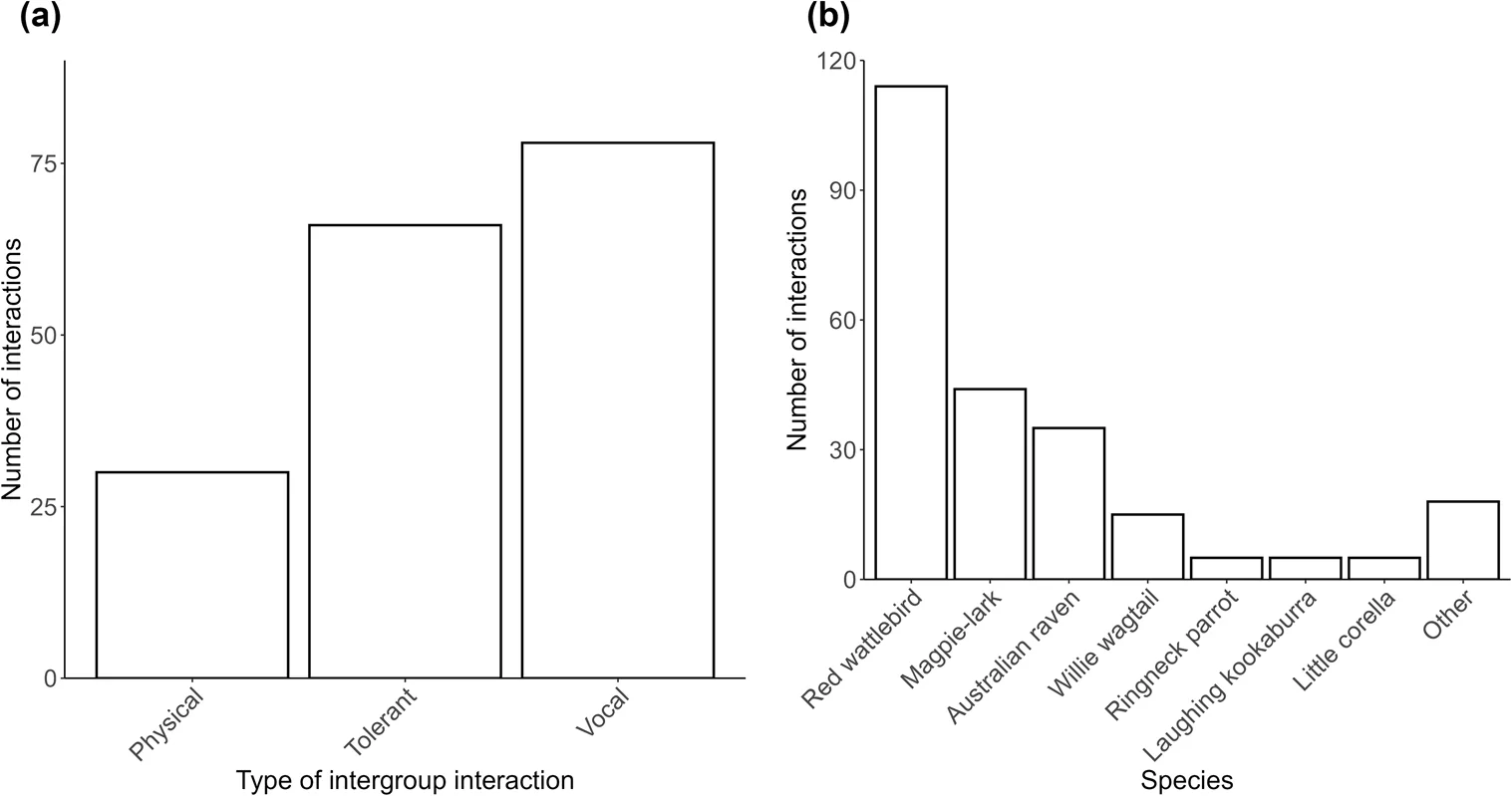
Histograms of (a) the number of each type of intergroup interaction and (b) the number of interspecific interactions with different species. ‘Other’ species interacted with include spotted turtle-doves (*Spilopelia chinensis*), galahs (*Eolophus roseicapilla*), white-faced herons (*Egretta novaehollandiae*), whistling kites (*Haliastur sphenurus*), common brushtail possums (*Trichosurus vulpecula*) and domestic cats (*Felis catus*).

Magpies interacted with other species on average 0.63 times an hour (± 0.06, *n* = 89 adult magpies). The most frequent type of interspecific interactions was those where aggression towards magpies was observed (66% of interspecific interactions involved magpies being swooped, chased or attacked by a heterospecific). The most common species that magpies interacted with were red wattlebirds (47.3% of interspecific interactions), followed by magpie larks (18.3% of interspecific interactions) and ravens (14.5% of interspecific interactions) (figure 2b). Sex did not affect how often individuals were involved in interspecific interactions (Mann–Whitney test, *w* = 1045, *p* = 0.648, *n* = 89 adult magpies).

### 3.4. Observational spatial memory/social cognition

Observational spatial memory score was not related to total intergroup or interspecific interaction rate, nor to the rate of any specific type of interaction (table 1; supplementary material, table S3). Observational spatial memory score was not affected by any measured proxy of motivation (supplementary material, table S4), with none of the candidate models outperforming the basic (null) model (table 1; supplementary material, tables S3 and S4).

**Table 1.** Model selection output for terms investigating the relationship between the rate of intergroup and interspecific interactions and performance on a social cognition task (observational spatial memory). (Notes: all models included group ID as a random term. Corrected Akaike's information criterion (AICc) values, ∆AICc and 95% confidence intervals (CI) are provided for each candidate model. For a full list of models tested, including different types of intergroup and interspecific interactions, see the supplementary material, table S3. *n* = 56 magpies from 16 groups.)

model	AICc	ΔAICc	CI
average intergroup interaction length^a^	351.61	0	−0.17, 2.56
null model	352.15	0.54	12.27, 15.05
interspecific interaction rate^a^	352.98	1.37	−2.30, 0.53
intergroup interaction rate	354.18	2.57	−1.01, 1.78

a Although this model is within 2 AICc of the top model, we do not consider it in the top model set as it is also within 2 AICc of the basic model, and its CIs intersect 0.

### 3.5. General cognitive performance in the physical domain

PCP was not related to total intergroup or interspecific interaction rate, or any type of interaction rate, nor was it related to any of our measured proxies of motivation (table 2; supplementary material, tables S5 and S6).

**Table 2.** Model selection output for terms investigating the relationship between intergroup and interspecific interactions and PCP. (Notes: corrected Akaike information criterion (AICc) values, ∆AICc and 95% confidence intervals (CI) are provided for each candidate model. For a full list of models tested, including different types of intergroup and interspecific interactions, see the supplementary material, table S5. *n* = 37 birds from 12 groups.)

model	AICc	ΔAICc	CI
null model	130.42	0	−0.41, 0.41
interspecific interaction rate	132.51	2.09	−0.17, 0.66
average intergroup interaction length	132.74	2.32	−0.52, 0.32
intergroup interaction rate	132.83	2.41	−0.49. 0.35

### 3.6. Response to quantity discrimination playbacks

The difference in response of individuals to playback of three versus one extragroup conspecific was affected by intergroup interaction rate (table 3; figure 3). Individuals that experienced more intergroup interactions exhibited a greater difference in vigilance response between one and three callers, suggesting that they were better able to discriminate between different numbers of extragroup conspecific callers. Analysis of *n* = 16 birds (removing one bird with a high intergroup interaction rate of 1.30) is presented in the supplementary material, S5. Results do not differ with the removal of this data point (supplementary material, table S10).

**Table 3.** Top model set of candidate terms affecting the difference in vigilance response between three callers and one caller. (Notes: all models included group ID as a random term. The top model set includes all models within 2 AICc of the top model and the null model (intercept-only model) for comparison. Coefficient estimates ± s.e. and 95% confidence intervals (CI) are given below the top model set. For a list of all models tested, see the supplementary material, table S10. *n* = 17 birds from 11 groups.)

top models	AICc	ΔAICc	
intergroup interaction rate	167.70	0.00	
null model	172.19	4.49	
**parameter**	**estimate**	**s.e.**	**CI**
intergroup interaction rate	20.00	6.01	7.26, 32.51

**Figure 3.**
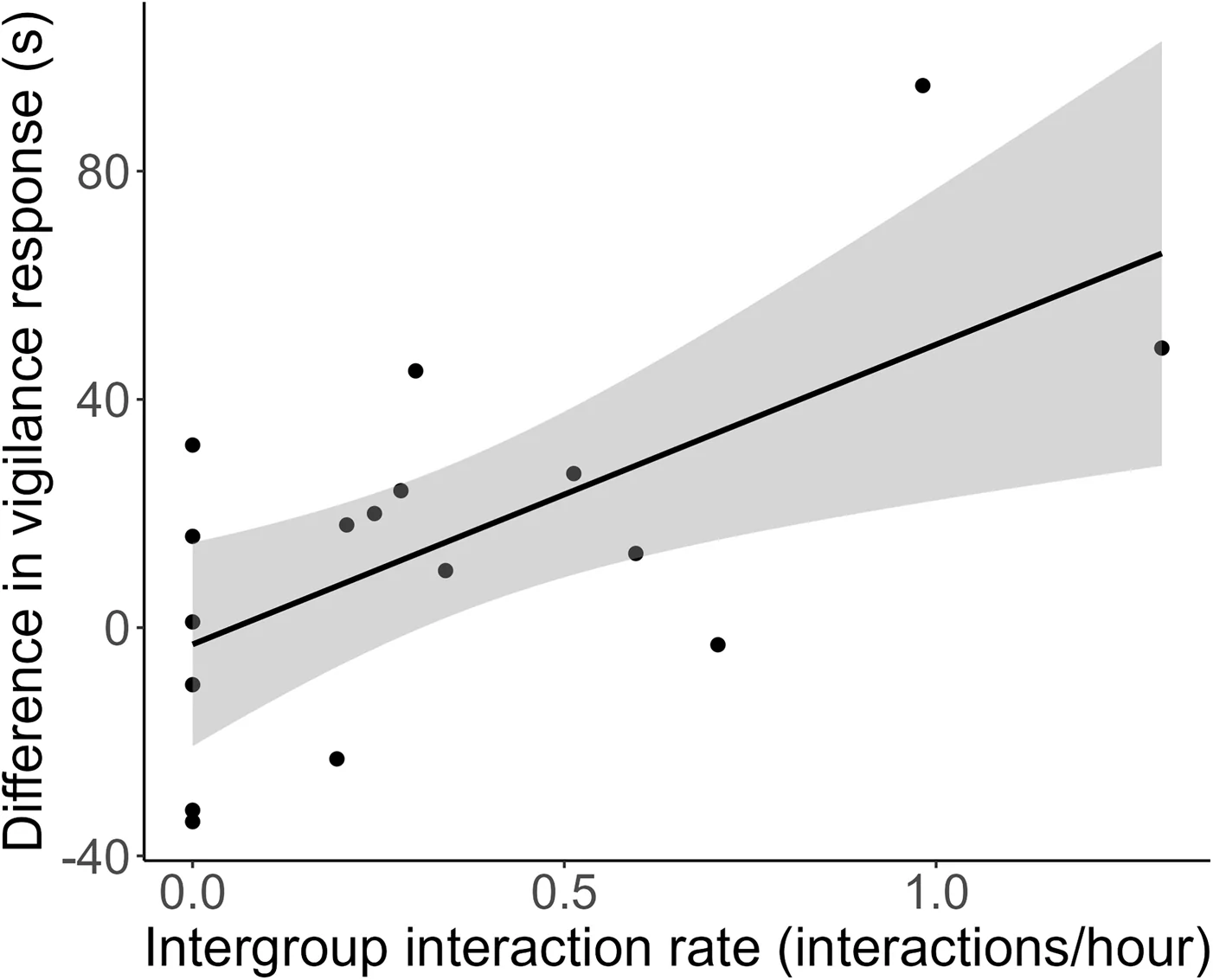
Difference in vigilance response to one versus three callers (calculated as time spent vigilant following three callers minus time spent vigilant following one caller) by intergroup interaction rate. Points represent raw data; shaded areas represent 95% confidence intervals; *n* = 17 individuals from 11 groups.

## 4. Discussion

In this study, we investigated whether extragroup sociality, specifically rates of intergroup and interspecific interactions, was related to cognitive performance in Western Australian magpies. In contrast to our hypothesis and that of Ashton *et al.* [29], we found no evidence that extragroup aspects of sociality were related to cognitive performance across four food-based cognitive tasks.

None of the measured aspects of intergroup sociality (rate of vocal interactions, physical aggressive interactions, tolerant interactions, total rate of intergroup interactions and average length of intergroup interactions) nor interspecific sociality (rate of magpie aggressive interactions, rate of interspecific aggressive interactions and total rate of interspecific interactions) were related to PCP or to individual performance on any of the cognitive tasks tested in this study (associative learning, spatial memory, quantity discrimination and observational spatial memory). This suggests that extragroup interactions may not be an important determinant of cognitive performance in this species. The only study to have previously investigated whether intergroup conflict was related to cognition, by Grueter [30], found a positive relationship between home-range size and endocranial volume across 104 primate species [30]. Interestingly, Grueter [30] also found a positive relationship between group size and endocranial volume, with group size being a stronger predictor of endocranial volume than home-range overlap, suggesting *intra*group sociality may be a more important determinant of cognition than *inter*group sociality in primates [30]. Previous research on magpies has identified positive relationships between multiple measures of intragroup sociality and cognitive performance [6,18,93,139]. Magpies typically have a high frequency of interaction with their group members, both when competing for resources and dominance, and cooperatively, when provisioning dependent young and coordinating territory defence [18,96,97]. Interactions with other groups or species probably occur less frequently than intragroup interactions, which may explain why we did not find a relationship between extragroup interactions and cognitive performance.

Alternatively, it may be that an unmeasured aspect of extragroup sociality is a more important determinant of cognitive performance in this species than those measured here. For example, individual magpies join many of the intergroup interactions that their social group engages in, potentially as a means of maintaining intragroup cohesion [140–142]. Therefore, the individual that initiates the intergroup interaction may face greater cognitive challenges than other individuals that join the contest later, as to initiate the contest this individual may have to assess both their own and the rival group and determine the cost versus benefit of initiating a conflict. The number of interactions an individual initiates may therefore more accurately represent the individual cognition–intergroup interaction relationship. Similarly, contest outcome may be a better predictor of cognitive performance [22]. However, correctly identifying the individual that initiated each intergroup interaction, or the group that won these interactions, was rarely feasible in our study, as magpies employ subtle cues to initiate and withdraw from contests.

Another, non-mutually exclusive explanation for our findings is that extragroup interactions may affect aspects of cognitive performance not measured in our study. Transitive inference, ‘the process of inferring the relationship between two items based on their shared relation with a third item’ [143–145], may be useful if magpies are observing the outcome of contests between neighbouring groups or individuals and can then infer from those interactions the potential cost of their own investment in such contests. Reversal learning, a common measure of cognitive and behavioural flexibility [11,146], could be useful if the outcomes of contests with extragroup individuals are variable, as it will allow individuals to readjust learnt associations between cues [29,38]. Highlighting the importance of reversal learning in contest scenarios, a recent study on captive domestic pigs (*Sus scrofa*) found that performance in a reversal learning task was positively related to contest outcome, with individuals that performed better on the task more likely to win a contest against an unfamiliar opponent [22]. Lee *et al.* [22] concluded that cognitive flexibility may help pigs to assess the behaviour of their rival and adjust their behaviour accordingly during a contest. More generally, the food-based cognitive tasks used in this study may not necessarily capture the cognitive abilities involved in extragroup interactions, which may be significantly more complex and involve simultaneous informational input from multiple sources. For example, extragroup interactions generally involve multimodal cues (i.e. visual, auditory and/or olfactory cues of the presence of an extragroup individual), while the tasks presented in our study present unimodal cues (visual and auditory cues for the cognitive tasks and playback experiment, respectively). Future studies should therefore expand upon the types of cognitive tasks used and cognitive skills tested in this study when investigating a potential relationship between extragroup interactions and cognition.

While performance in a battery of four food-based cognitive tasks was not related to extragroup interactions, we identified a relationship between intergroup interaction rate and response to a quantity discrimination playback experiment. Specifically, individuals that were involved in more intergroup interactions were better able to discriminate between playback of one and three callers, an ability likely to involve quantity discrimination abilities [43,46,108]. The ability to assess and discriminate between vocal cues of individuals is beneficial in contest scenarios [147,148], particularly for territorial species that vocalize to defend territories, and hence this may be a more ecologically relevant test of quantity discrimination abilities than a task assessing quantity discrimination between different numbers of food items. The relationship seen between intergroup interaction rates and vigilance response to playbacks probably arises owing to experience; birds that experience more interactions with extragroup conspecifics (and therefore hear and respond to the songs of more extragroup conspecifics) may have honed their ability to discriminate between the songs of one and multiple callers. This is probably beneficial for individuals when assessing the RHP of other groups, before deciding whether to enter into a contest with them, as numerical advantage may have a significant influence on contest outcome [149,150]. It is important to note that, owing to our presentation of natural (rather than artificially generated or altered) territorial songs, we cannot rule out that magpies may have been using non-numerical cues (such as song length or amplitude, both of which differed between one- and three-caller songs) to discern the number of callers and inform their response to playback. However, these cues would be present in natural intergroup interactions and hence are ecologically relevant in contest scenarios for this species [127]. Furthermore, it is likely that birds use multiple cognitive abilities (both social and physical) while perceiving, processing, assessing and responding to playback of extragroup individuals, and hence response to these playbacks may not solely be a reflection of quantity discrimination abilities, but rather of several cognitive abilities used in this scenario. Nevertheless, this novel finding directly links naturally occurring intergroup interactions with playbacks simulating differing numbers of callers, indicating the importance of quantity discrimination abilities in the context of intergroup contests [43,46,47,151].

In this study, we tested the relationship between adult social context and adult cognitive performance, an approach taken by several studies on animal cognition [18,94,152]. For adult extragroup interactions and adult cognitive performance to be related, one of two assumptions must be met. The first assumption is that both cognition and extragroup interaction rates are stable and repeatable in the long term, such that cognitive performance and extragroup interaction rates measured in adulthood reflect those experienced during development. The second assumption is that extragroup interaction rates and cognitive performance are not stable and instead covary over time. For example, cognitive performance may vary over time in response to the current (or recent) social context experienced by an individual, as has been found to be the case in pheasants (*Phasianus colchicus*) [153] and Guinea baboons (*Papio papio*) [154]. Previous research on this species has identified long-term repeatability in performance on certain cognitive tasks (associative learning, reversal learning and quantity discrimination), but not on others (spatial memory and inhibitory control) [127,155], suggesting that the stability of cognitive performance over time may vary between cognitive tasks. Whether extragroup interaction rates experienced by magpies are repeatable in the long term could not be tested in the present study; however, it may be the case in this population owing to the stable size and composition of magpie groups, the low levels of dispersal documented in our population and the relative permanence of territory borders [82,96,98]. A longitudinal study of cognition over both the developmental and adult periods in relation to social context would be needed to confirm if extragroup interactions influence the development of cognitive phenotypes.

## 5. Conclusion

In this study, we investigated whether extragroup aspects of sociality were related to cognitive performance in Western Australian magpies. We found that neither intergroup nor interspecific interactions were related to the cognitive performance of magpies across four ecologically relevant tasks; however, intergroup interaction rate was related to the ability of individuals to discriminate between different numbers of conspecific callers. The influence of extragroup sociality on cognition has thus far been largely overlooked, with our study being one of only two empirical studies to our knowledge to investigate this relationship, and as such further research on other social species is needed to understand the potential impact of extragroup sociality on cognition.

## Supplementary Material



## Data Availability

Data and code used in analysis have been deposited and are available through figshare [156]. Supplementary material is available online [157].
