# Supplementary material for: Extragroup interactions are not related to cognitive performance in a group-living passerine
Source: R Soc Open Sci. 2026 Oct 28;13(10):260901. doi: 10.1098/rsos.260901 (PMC13714422; doi:10.1098/rsos.260901)
Supplement: Supplementary file 1 [file rsos.260901.zip › rsos.260901/rsos260901_si_001.docx]

**Extragroup interactions are not related to cognitive performance in a group-living passerine**

Blackburn, G., Ashton, B. J., Thornton, A., & Ridley, A. R. Extragroup interactions are not related to cognitive performance in a group-living passerine. *Royal Society Open Science.* 10.1098/rsos.260901

***SUPPLEMENTARY MATERIAL***

**Section 1 – General cognitive performance in the physical domain (PCP)**

**Table S1**. Kendall rank correlation matrix of performance in all physical cognitive tasks.

|  |  | Spatial Memory | Associative learning |
| --- | --- | --- | --- |
| Associative learning | rs  P  N | 0.208  0.09  37 |  |
| Quantity discrimination | rs  P  N | 0.177  0.140  37 | 0.191  0.115  37 |

**Table S2.** Results of PCA for individuals that completed the three physical cognitive tasks (*N* = 37). Loadings and percentage of total variance explained for each principal component with an eigenvalue > 1 are shown. Loadings >0.5 are in bold.

| **Task** | **PC1** |
| --- | --- |
| Spatial memory | **0.765** |
| Associative learning | **0.776** |
| Quantity discrimination | **0.685** |
| Eigenvalue | 1.657 |
| % total variance explained | 55.25% |

| **Model** | **AICc** | **ΔAICc** | **CI** |
| --- | --- | --- | --- |
| Average intergroup interaction length^1^ | 351.61 | 0 | -0.17, 2.56 |
| *Null model* | *352.15* | *0.54* | *12.27, 15.05* |
| Magpie aggressive rate (interspecific)^1^ | 352.16 | 0.55 | -2.46, 0.30 |
| Average intergroup interaction length*Sex (male)^1^ | 352.90 | 1.29 | -5.31, 0.06 |
| Interspecific interaction rate^1^ | 352.98 | 1.37 | -2.30, 0.53 |
| Vocal rate (intergroup) | 353.75 | 2.14 | -0.79, 2.00 |
| Interspecific aggressive rate (interspecific) | 354.15 | 2.54 | -1.83, 1.01 |
| Intergroup interaction rate | 354.18 | 2.57 | -1.01, 1.78 |
| Distance to task | 354.25 | 2.64 | -1.06, 1.73 |
| Neophobia | 354.28 | 2.67 | -1.08, 1.71 |
| Physical aggressive rate (intergroup) | 354.36 | 2.75 | -1.16, 1.64 |
| Sex | 354.43 | 2.82 | -2.50, 3.05 |
| Tolerance rate (intergroup) | 354.47 | 2.86 | -1.44, 1.36 |

**Section 2 - Analysis of the effects of extragroup interactions on observational spatial memory performance (a measure of social cognition)**

**Table S3.** Full model selection output for candidate terms affecting observational spatial memory performance. All models included group ID as a random term. Corrected Akaike information criterion (AICc), ∆AICc, and 95% confidence intervals (CI) are provided for each candidate model. *N =* 56 birds from 16 groups.

^1^Although within 2AICc of the top model, not considered in top model set as within 2AICc of the basic model, and confidence intervals intersect 0.

**Table S4.** Full model selection output for candidate terms affecting observational spatial memory performance on a subset of individuals with foraging focals and body mass measurements. All models included group ID as a random term. Corrected Akaike information criterion (AICc), ∆AICc, and 95% confidence intervals (CI) are provided for each candidate model. Only candidate models within 2 AICc of the top model, and with predictors whose 95% CI did not intersect zero were included in the top model set and are highlighted in bold. *N =* 44 birds from 14 groups.

| **Model** | **AICc** | **∆AICc** | **CI** |
| --- | --- | --- | --- |
| **Average intergroup interaction length** | **270.18** | **0.00** | **0.34, 3.16** |
| Vocal rate (intergroup)^1^ | 271.73 | 1.55 | -0.23, 3.17 |
| *Null model* | 272.24 | 2.06 | 12.07,15.59 |
| Physical aggressive rate (intergroup) | 272.91 | 2.73 | -3.23, 0.63 |
| Intergroup interaction rate | 273.76 | 3.58 | -0.86, 2.59 |
| Tolerance rate (intergroup) | 273.90 | 3.72 | -0.87, 2.32 |
| Body mass | 274.01 | 3.83 | -2.21, 0.91 |
| Foraging efficiency | 274.02 | 3.84 | -0.88, 2.12 |
| Neophobia | 274.06 | 3.88 | -0.89, 2.06 |
| Distance to task | 274.13 | 3.95 | -0.92, 2.03 |
| Magpie aggressive rate (interspecific) | 274.33 | 4.15 | -1.90, 1.03 |
| Foraging effort | 274.45 | 4.27 | -1.12, 1.83 |
| Interspecific interaction rate | 274.56 | 4.38 | -1.74, 1.24 |
| Interspecific aggressive rate (interspecific) | 274.63 | 4.45 | -1.63, 1.32 |
| Sex | 274.65 | 4.47 | -3.10, 2.67 |

^1^Although within 2AICc of the top model, not considered in top model set as within 2AICc of the basic model, and confidence intervals intersect 0.

**Section 3 - Analysis of the effects of extragroup interactions on general cognitive performance in the physical domain (PCP)**

**Table S5.** Full model selection output for candidate terms affecting PCP. All models included group ID as a random term. Corrected Akaike information criterion (AICc), ∆AICc, and 95% confidence intervals (CI) are provided for each candidate model. *N =* 37 birds from 12 groups.

| **Model** | **AICc** | **ΔAICc** | **CI** |
| --- | --- | --- | --- |
| Magpie aggressive rate (interspecific)^1^ | 129.85 | 0 | -0.03, 0.77 |
| *Null model* | *130.42* | *0.57* | *-0.41, 0.41* |
| Interspecific interaction rate^1^ | 131.59 | 1.74 | -0.17, 0.66 |
| Foraging efficiency | 132.21 | 2.36 | -0.23, 0.60 |
| Sex | 132.68 | 2.83 | -1.05, 0.61 |
| Interspecific aggressive rate (interspecific) | 132.72 | 2.87 | -0.52, 0.32 |
| Average intergroup interaction length | 132.74 | 2.89 | -0.52, 0.32 |
| Neophobia | 132.77 | 2.92 | -0.33, 0.21 |
| Vocal rate (intergroup) | 132.81 | 2.96 | -0.50, 0.34 |
| Intergroup interaction rate | 132.83 | 2.98 | -0.49. 0.35 |
| Foraging effort | 132.90 | 3.05 | -0.47, 0.37 |
| Tolerance rate (intergroup) | 132.90 | 3.05 | -0.46, 0.38 |
| Physical aggressive rate (intergroup) | 132.92 | 3.07 | -0.45, 0.39 |

^1^Although within 2AICc of the top model, not considered in top model set as within 2AICc of the basic model.

**Table S6.** Full model selection output for candidate terms affecting PCP for a subset of individuals for whom we had body mass measurements. All models included group ID as a random term. Corrected Akaike information criterion (AICc), ∆AICc, and 95% confidence intervals (CI) are provided for each candidate model. *N =* 36 birds from 12 groups.

| **Model** | **AICc** | **ΔAICc** | **CI** |
| --- | --- | --- | --- |
| Magpie aggressive rate (interspecific)^1^ | 126.26 | 0.00 | -0.01, 1.04 |
| *Null model* | *128.02* | *1.76* | *-0.42, 0.44* |
| Interspecific interaction rate | 128.89 | 2.63 | -0.14, 0.70 |
| Foraging efficiency | 129.90 | 3.64 | -0.25, 0.61 |
| Sex | 130.24 | 3.98 | -1.09, 0.60 |
| Interspecific aggressive rate (interspecific) | 130.34 | 4.08 | -0.53, 0.33 |
| Average intergroup interaction length | 130.39 | 4.13 | -0.52, 0.34 |
| Neophobia | 130.42 | 4.16 | -0.51, 0.35 |
| Vocal rate (intergroup) | 130.44 | 4.18 | -0.51, 0.35 |
| Intergroup interaction rate | 130.45 | 4.19 | -0.50, 0.36 |
| Body mass | 130.52 | 4.26 | -0.39, 0.47 |
| Tolerance rate (intergroup) | 130.52 | 4.26 | -0.47, 0.39 |
| Physical aggressive rate (intergroup) | 130.53 | 4.27 | -0.47, 0.39 |
| Foraging effort | 130.54 | 4.28 | -0.46, 0.40 |

^1^Although within 2AICc of the top model, not considered in top model set as within 2AICc of the basic model, and confidence intervals intersect 0.

**Section 4 - Analysis of the effects of extragroup interactions on separate performance in the spatial memory, associative learning, and quantity discrimination** **tasks.**

*Statistical analysis*

To investigate how extragroup interactions were related to cognitive performance, and to determine whether these interactions affected performance on the three physical cognitive tasks separately (rather than GCP), we fitted LMMs and GLMMs (Generalised Linear Mixed Models) with spatial memory score, associative learning score, and quantity discrimination score as the response variables. LMMs investigating the factors affecting spatial memory and quantity discrimination had normal distributions, while GLMMs investigating factors affecting associative learning had a negative binomial distribution. All models included group identity as a random term. Intergroup interaction predictors included vocal interaction rate (calculated as the number of vocal intergroup interactions per observation hour), physical aggressive interaction rate (calculated as the number of physical aggressive intergroup interactions per observation hour), and tolerance interaction rate (calculated as the number of tolerant intergroup interactions per observation hour), as well as a combined predictors of total intergroup interaction rate (calculated as the total number of intergroup interactions per observation hour), and the average length of intergroup interactions (in seconds). Interspecific interaction predictors included magpie aggressive interaction rate (calculated as the number of interspecific interactions where magpies were aggressive to other species per observation hour), interspecific interaction rate (calculated as the number of interspecific interactions where other species were aggressive to magpies per observation hour), and a combined predictor of total interspecific interaction rate (calculated as the total number of interspecific interactions per observation hour) as explanatory terms. Predictors were tested for collinearity using the variance inflation factor (VIF), and predictors that were correlated (had a VIF > 2, considered a high level of correlation (Fox and Monette 1992), e.g. tolerance interaction rate and vocal interaction rate), were not included together in the same model.

**Table S7.** Full model selection output for candidate terms affecting spatial memory performance. All models included group ID as a random term. Corrected Akaike information criterion (AICc), ∆AICc, and 95% confidence intervals (CI) are provided for each candidate model. *N =* 56 birds from 16 groups.

| **Model** | **AICc** | **ΔAICc** | **CI** |
| --- | --- | --- | --- |
| Magpie aggressive rate (interspecific)^1^ | 373.74 | 0.00 | -0.28, 3.05 |
| *Null model* | *374.03* | *0.29* | *10.99, 14.37* |
| Average intergroup interaction length | 375.96 | 2.22 | -2.24, 1.15 |
| Interspecific interaction rate | 375.99 | 2.25 | -1.18, 2.22 |
| Interspecific aggressive rate (interspecific) | 376.02 | 2.28 | -2.19, 1.20 |
| Neophobia | 376.11 | 2.37 | -1.28, 2.12 |
| Sex | 376.14 | 2.40 | -4.15, 2.58 |
| Vocal rate (intergroup) | 376.28 | 2.54 | -1.93, 1.48 |
| Intergroup interaction rate | 376.33 | 2.59 | -1.84, 1.57 |
| Tolerance rate (intergroup) | 376.35 | 2.61 | -1.72, 1.68 |
| Physical aggressive rate (intergroup) | 376.35 | 2.61 | -1.72, 1.68 |

^1^Although within 2AICc of the top model, not considered in top model set as within 2AICc of the basic model, and confidence intervals intersect 0.

**Table S8.** Full model selection output for candidate terms affecting associative learning performance. All models included group ID as a random term. Corrected Akaike information criterion (AICc), ∆AICc, and 95% confidence intervals (CI) are provided for each candidate model. *N =* 51 birds from 16 groups.

| **Model** | **AICc** | **ΔAICc** | **CI** |
| --- | --- | --- | --- |
| Interspecific interaction rate^1^ | 346.68 | 0.00 | -0.01, 0.21 |
| *Null model* | *348.02* | *1.34* | *2.73, 2.98* |
| Shade rewarded^1^ | 348.45 | 1.77 | -0.06, 0.40 |
| Interspecific aggressive rate (interspecific)^1^ | 348.52 | 1.84 | -0.03, 0.19 |
| Tolerance rate (intergroup) | 348.74 | 2.06 | -0.19, 0.04 |
| Magpie aggressive rate (interspecific) | 348.90 | 2.22 | -0.04, 0.19 |
| Average intergroup interaction length | 349.06 | 2.37 | -0.18, 0.05 |
| Physical aggressive rate (intergroup) | 349.28 | 2.60 | -0.05, 0.18 |
| Vocal rate (intergroup) | 349.73 | 3.05 | -0.16, 0.07 |
| Intergroup interaction rate | 349.88 | 3.20 | -0.16, 0.07 |
| Sex | 349.97 | 3.29 | -0.17, 0.33 |
| Colour rewarded (Blue)  Green  Grey  Pink  Purple  Red | 352.27 | 5.59 | -0.80, 0.06  -0.19, 0.62  -0.86, 0.73  -0.54, 0.06  -0.79, 0.41 |

^1^Although within 2AICc of the top model, not considered in top model set as within 2AICc of the basic model, and confidence intervals intersect 0.

**Table S9.** Full model selection output for candidate terms affecting quantity discrimination performance. All models included group ID as a random term. Corrected Akaike information criterion (AICc), ∆AICc, and 95% confidence intervals (CI) are provided for each candidate model. *N =* 47 birds from 13 groups.

| **Model** | **AICc** | **ΔAICc** | **CI** |
| --- | --- | --- | --- |
| *Null model* | *282.02* | *0.00* | *13.61, 17.15* |
| Physical aggressive rate (intergroup)^1^ | 283.10 | 1.08 | -2.30, 0.58 |
| Interspecific aggressive rate (interspecific)^1^ | 283.72 | 1.70 | -1.87, 0.75 |
| Neophobia | 284.10 | 2.08 | -1.04, 1.90 |
| Sex | 284.24 | 2.22 | -2.90, 1.86 |
| Intergroup interaction rate | 284.27 | 2.25 | -2.01, 1.34 |
| Tolerance rate (intergroup) | 284.31 | 2.29 | -1.37, 1.93 |
| Magpie aggressive rate (interspecific) | 284.37 | 2.35 | -1.12, 1.41 |
| Vocal rate (intergroup) | 284.37 | 2.35 | -1.76, 1.40 |
| Average intergroup interaction length | 284.37 | 2.35 | -1.87, 1.50 |
| Interspecific interaction rate | 284.40 | 2.38 | -1.37, 1.19 |

^1^Although within 2AICc of the top model, not considered in top model set as within 2AICc of the basic model, and confidence intervals intersect 0.

***Section 5 - Quantity discrimination*** ***playbacks and intergroup interactions***

| **Model** | **AICc** | **ΔAICc** |
| --- | --- | --- |
| **Intergroup interaction rate** | **167.70** | 0.00 |
| *Null model* | *172.19* | *4.49* |
| Sex | 174.80 | 7.10 |

**Table S10.** Full model selection output for candidate terms affecting the difference in vigilance response between three callers and one caller. All models included group ID as a random term. Corrected Akaike information criterion (AICc) and ∆AICc are provided for each candidate model. Only models within 2 AICc of the top model, and with predictors whose 95% confidence intervals did not intersect zero were included in the top model set and are highlighted in bold. *N =* 17 birds from 11 groups.

| **Predictor** | **AICc** | **∆AICc** |
| --- | --- | --- |
| *Null model* | *84.13* | *0.00* |
| Intergroup interaction rate | 86.42 | 2.29 |
| Sex | 87.50 | 3.37 |

**Table S11.** Full model selection output for candidate terms affecting the difference in song response between three callers and one caller. All models included group ID as a random term. Corrected Akaike information criterion (AICc) and ∆AICc are provided for each candidate model. Only models within 2 AICc of the top model, and with predictors whose 95% confidence intervals did not intersect zero were included in the top model set. *N =* 17 birds from 11 groups.

***Section 6 - Quantity discrimination*** ***playbacks and intergroup interactions, N = 16***

| **Model** | **AICc** | **ΔAICc** |
| --- | --- | --- |
| **Intergroup interaction rate** | **158.91** | **0.00** |
| *Null model* | *162.41* | *3.49* |
| Sex | 165.61 | 6.70 |

**Table S12.** Full model selection output for candidate terms affecting the difference in vigilance response between three callers and one caller. All models included group ID as a random term. Corrected Akaike information criterion (AICc) and ∆AICc are provided for each candidate model. Only models within 2 AICc of the top model, and with predictors whose 95% confidence intervals did not intersect zero were included in the top model set and are highlighted in bold. *N =* 16 birds from 11 groups.

**Table S13.** Top model set of candidate terms affecting the difference in vigilance response between three callers and one caller. All models included group ID as a random term. The top model set includes all models within 2 AICc of the top model and the null model (intercept-only model) for comparison. Coefficient estimates ± S.E. and 95% confidence intervals (CI) are given below the top model set. For a list of all models tested, see Supplementary Materials Table S12. *N =* 16 birds from 11 groups

| **Top Models** | **AICc** | **ΔAICc** |  |
| --- | --- | --- | --- |
| Intergroup interaction rate | 158.91 | 0.00 |  |
| *Null model* | *162.41* | *3.49* |  |
| **Parameter** | **Estimate** | **S. E** | **C.I** |
| Intergroup interaction rate | 19.42 | 6.23 | 6.24, 32.40 |

**Figure S1.** Difference in vigilance response to one versus three callers (calculated as time spent vigilant following three callers minus time spent vigilant following one caller) by intergroup interaction rate. Points represent raw data; shaded areas represent 95% confidence intervals. *N* = 16 individuals from 11 groups.

**Table S14.** Full model selection output for candidate terms affecting the difference in song response between three callers and one caller. All models included group ID as a random term. Corrected Akaike information criterion (AICc) and ∆AICc are provided for each candidate model. Only models within 2 AICc of the top model, and with predictors whose 95% confidence intervals did not intersect zero were included in the top model set. *N =* 16 birds from 11 groups.

| **Predictor** | **AICc** | **∆AICc** |
| --- | --- | --- |
| *Null model* | *80.64* | *0.00* |
| Intergroup interaction rate | 83.08 | 2.44 |
| Sex | 84.09 | 3.45 |
